# Rare Presentation of Small Bowel Obstruction Secondary to Intestinal Leiomyoma in a Patient with Crohn's Disease

**DOI:** 10.1155/2023/8008712

**Published:** 2023-03-03

**Authors:** Benjamin J. Behers, Brett M. Behers, Ryan C. Perez, Kabir Grewal, Joe Cyriac, Joanne Makar, Russell W. Novak

**Affiliations:** ^1^Florida State University College of Medicine, Tallahassee, FL, USA; ^2^University of South Florida College of Medicine, Tampa, FL, USA; ^3^General Surgery, Sarasota Memorial Hospital, Sarasota, FL, USA

## Abstract

Small bowel obstructions (SBOs) are surgical emergencies that can occur with mechanical blockage of the intestinal lumen. These blockages are most commonly caused by adhesions or hernias, but can also result from intestinal neoplasms. This case report documents the rare occurrence of SBO due to a submucosal leiomyoma. This case was complicated by the patient's longstanding history of Crohn's disease, which has a considerable overlap in symptomatology with SBOs. This may have resulted in a delay in the patient's diagnosis. Leiomyomas should always be considered as a potential, albeit rare, cause of SBO.

## 1. Introduction

Small bowel obstructions (SBOs) are surgical emergencies that can occur with mechanical blockage of the intestinal lumen. These blockages are most commonly caused by adhesions or hernias [1]. Other common etiologies include inflammatory strictures, foreign bodies, and neoplasms [1, 2]. Despite comprising a vast majority of the gastrointestinal tract, the small bowel only accounts for approximately 3% of all its neoplasms, with an estimated 10% of these representing benign lesions [3, 4]. Adenomas are the most common benign small bowel tumor, but over 40 other types exist, including leiomyomas [5]. A common feature of small bowel tumors is their insidious onset, due to slow growth and non-specific symptoms, including abdominal pain, nausea, and weight loss [4, 5]. As a result, many are found incidentally or never at all. This case documents a rare presentation of SBO due to a submucosal leiomyoma in a patient with underlying Crohn's disease.

### 1.1. Case Presentation

A 54-year-old male presented to our outpatient general surgery office on a referral from his gastroenterologist. The patient reported a 2-month history of worsening abdominal pain, bloating, and emesis, with an accompanying 50-pound weight loss. His presentation raised concern for bowel obstruction.

The patient's medical history was notable for a 20-year history of poorly controlled small bowel Crohn's disease. Thirteen years prior to this case, he had developed a perirectal abscess that required drainage. Five years prior, he had developed a perirectal fistula that required placement of a seton drain. His current medications included omeprazole, budesonide, and mesalamine. He had also recently started the induction phase of adalimumab for Crohn's treatment. A recent capsule endoscopy noted ulceration, erythema, and edema of the distal small bowel, findings consistent with his active Crohn's disease.

On physical examination, the patient's vital signs were within reference range, with a blood pressure of 120/72 mmHg, a pulse of 71 BPM, a temperature of 97.6°F, and a body mass index of 22.7 kg/m^2^. His general appearance showed a middle-aged Caucasian male in obvious discomfort. He had noticeable pallor, with sunken eyes and dry, cracked lips. His abdomen was soft but distended; with notable tympany but no pain on percussion. He did not have any abdominal scars, peritoneal findings, or hernias.

A computed tomography (CT) report from an outside facility described markedly dilated small bowel loops, consistent with a high-grade SBO. There was no transition point readily appreciated. A plan was developed for an exploratory laparotomy to search for and remove any sources of obstruction. The risks, benefits, and alternatives were discussed at length, with the patient expressing understanding and agreeing to the procedure. Due to his dehydration, the patient was admitted to the hospital to undergo hydration and bowel prep prior to having surgery 2 days later.

**Figure 1:**
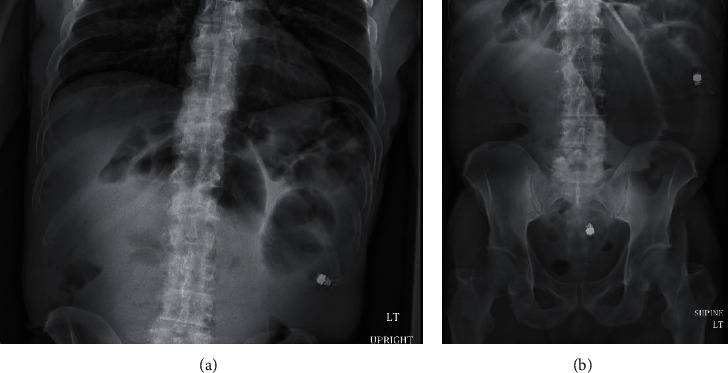
Upright (a) and supine (b) abdominal X-rays taken on admission showing dilated loops of small bowel in the central and left abdomen.

Upon hospital admission, upright and supine abdominal X-rays were obtained to verify the obstruction. Dilated loops of small bowel with air-fluid levels were noted throughout the central and left abdomen, the largest of which measured 6.7 cm. These findings further confirmed the suspected SBO (Figure 1).

Laboratory findings on admission were notable for a mild anemia with a hemoglobin level of 11.5 g/dL (reference range: 14.0–17.5 g/dL) and a hematocrit of 36.6% (reference range: 40.0–52.0%). His normal white blood cell count, in addition to his normal temperature and the lack of peritoneal signs on physical exam, lowered the concern for an acute infection or flare of his Crohn's disease. Despite the patient's recent weight loss and dehydrated status, electrolytes and renal function were within reference limits.

**Figure 2:**
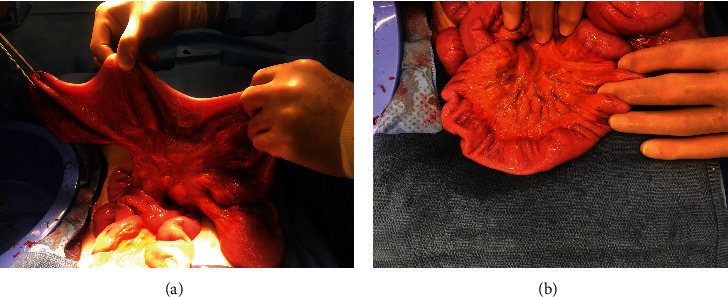
Intraoperative pictures documenting markedly dilated loops of small bowel proximal to the tumor (a) and normal loops downstream (b).

On day 3 of his hospitalization, the exploratory laparotomy was performed. Upon entrance into the peritoneum, markedly dilated loops of small bowel were found proximal to a tumor of the small bowel wall in the jejunum. This tumor significantly narrowed the lumen but did not fully obstruct it. The small bowel appeared normal-sized distal to this mass (Figure 2). The segment of small bowel containing the tumor was resected and sent to pathology for analysis.

**Figure 3:**
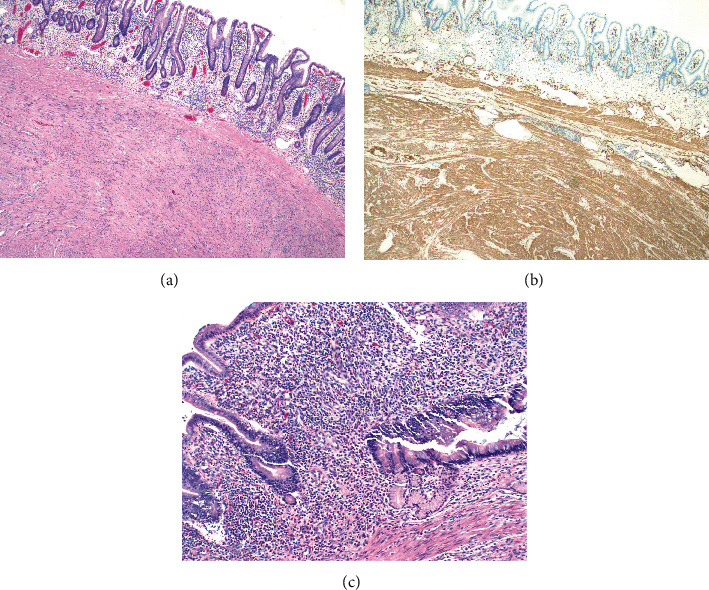
Histology slides from the specimen documenting: (a) small bowel with a submucosal leiomyoma on a hematoxylin and eosin stain magnified 40×; (b) smooth muscle actin positivity on immunohistochemistry magnified 40×; and (c) moderate-to-severe active chronic enteritis on a hematoxylin and eosin stain magnified 100×.

Pathology results determined the mass to be a 3.0 × 2.7 × 2.5 cm submucosal leiomyoma. Immunostains of the mass were positive for smooth muscle actin (SMA) and desmin; along with non-specific reactivity for DOG-1 and no specific reactivity for S-100, CD117, HMB, or CKAE1/3. Further, moderate-to-severe active chronic enteritis was noted (Figure 3).

The patient tolerated the procedure well and had minimal postoperative complications. His ileus was resolved by postoperative day 5, with subsequent removal of his nasogastric tube. He was able to tolerate clear fluids that day and progressed to a full liquid diet the following day. He remained on patient-controlled analgesia with hydromorphone and intravenous antibiotics of cefazolin and metronidazole until postoperative day 6. A low-residue diet was resumed on postoperative day 7. His discharge occurred on postoperative day 7, resulting in a total hospitalization length of 10 days. He was discharged on as-needed hydrocodone, with additional recommendations for protein supplements, vitamin supplementation, and probiotics.

He returned to the outpatient general surgery office 19 days after the procedure. His incision site had closed, with some residual scabbing but no erythema or signs of infection. He reported he was doing very well and was now tolerating solid foods without emesis for the first time in months. He had achieved a 20-pound weight gain since his initial presentation. His staples were removed on this visit.

Upon following up with his gastroenterologist, the patient continued to report no abdominal pain, nausea, or vomiting. He was advised to continue the adalimumab, in hopes of better future control of his Crohn's disease, as well as the rest of his current medications mentioned. Further, it was recommended that he increase his intake of dietary fiber and adhere to strict avoidance of smoking, smoke exposure, and non-steroidal anti-inflammatory medications (NSAIDs). His surveillance plan includes bi-annual assessments of inflammatory markers, such as erythrocyte sedimentation rate (ESR) and C-reactive protein (CRP), to monitor his disease state. Further, he will continue regular screening colonoscopies and capsule endoscopies, with magnetic resonance enterography in the event that his small bowel mucosa cannot be directly visualized. Despite having the colonoscopy and capsule endoscopy this year, these will be repeated next year in light of this event. His future screening schedule will be assessed at that time.

## 2. Discussion

Here, we highlight the diagnostic challenges that submucosal leiomyomas can pose, especially in patients with concurrent inflammatory bowel disease. Our patient's symptoms had an insidious onset that occurred over several months, and several imaging modalities (X-ray, CT, and capsule endoscopy) failed to identify the mass prior to its intraoperative discovery. This was also an atypical presentation of SBO, as they usually present with acute, rapidly progressive symptoms and have an identifiable transition point on imaging. Further, complicating the case was the overlap between our patient's symptoms and previous flares of his Crohn's disease, which also included nausea, vomiting, abdominal pain, and weight loss.

While SBOs are a known complication of Crohn's disease, these blockages are usually the result of inflammatory strictures or fistulas that narrow the intestinal lumen [6]. However, several case reports have described obstructions resulting from intraluminal tumors in patients with Crohn's disease, with adenocarcinomas being the most common [6]. This case report suggests that leiomyomas should also be considered on the differential of SBO.

Leiomyomas are benign tumors that originate from smooth muscle cells derived from mesenchymal tissue [7]. Although they are most commonly found in the uterus, leiomyomas can occur wherever smooth muscle is present, including along the entire length of the intestine. Within the small bowel, leiomyomas are predominantly found in the jejunum [7]. These tumors are often asymptomatic and are generally found incidentally through imaging or during unrelated abdominal procedures. The most common symptomatic presentation results from hemorrhage, which can occur as the tumor outgrows its blood supply, resulting in ulceration and necrosis [7]. Intussusception is a less common presentation, wherein the leiomyoma acts as the lead point [7].

It is rare for leiomyomas to present as an obstruction, and there are only a handful of case reports detailing this phenomenon. One such report followed a previously healthy 87-year-old female who presented with a 1-day history of worsening abdominal pain and distention, obstipation, and vomiting [8]. Her CT scan revealed a transition point with a calcified intraluminal mass, and an emergency laparotomy was performed to remove the mass, followed by a right hemicolectomy with an end-to-end ileocolic anastomosis [8]. The acute onset of symptoms and positive imaging results described in this case was in line with the typical SBO presentation, unlike the case we report.

The pathogenesis of leiomyoma formation is not well understood. Most of the existing literature has focused on uterine leiomyomas, which are both more prevalent and more likely to be symptomatic in comparison to intestinal leiomyomas [9]. Several genetic polymorphisms have been associated with uterine leiomyoma formation, namely in genes encoding receptors for growth factors and androgens [10]. Leiomyoma development has also been linked to increased levels of cellular mediators, including insulin-like growth factor II (IGF-II), vascular endothelial growth factor (VEGF), and numerous other regulators of inflammation and growth [10]. This suggests an association between uterine leiomyoma formation and states of chronic inflammation, in which these mediators are chronically elevated [10, 11]. Future research could explore whether these polymorphisms and cellular mediators are also associated with intestinal leiomyoma formation. Further, researchers could explore the overlap between these factors and the pathophysiology of Crohn's disease. For example, associations have already been discovered between inflammatory bowel diseases and elevated levels of VEGF [12]. If a relationship does exist between leiomyomas and Crohn's disease, this patient could be at risk for recurrence and further intervention outside of his current screening plan may be indicated. However, given the slow rate of growth of small bowel tumors, including leiomyomas, and no other areas of concern identified during the laparotomy, his prognosis seems favorable.

While no direct links have been made in the literature between Crohn's disease and leiomyoma formation, Crohn's has been identified as a risk factor for the formation of adenocarcinomas, which have a similar potential to obstruct the intestinal lumen [13, 14]. Crohn's has also been linked to the formation of inflammatory fibroid polyps, which are benign mesenchymal tumors of the gastrointestinal tract linked to states of chronic inflammation [15]. Several case reports have described the discovery of these polyps in patients with Crohn's disease after they resulted in intussusception of the small bowel [16–20]. The similarities between leiomyomas and inflammatory fibroid polyps, as they are both benign mesenchymal tumors that share many cellular markers, further support a possible association between Crohn's disease and leiomyomas.

## 3. Conclusions

This case report describes the clinical presentation of SBO secondary to a submucosal leiomyoma that formed in a patient with a history of Crohn's disease. Leiomyomas should always be considered as a potential, albeit rare, cause of SBO. Diagnosis can be difficult for clinicians, as evidenced by the lack of detection from prior capsule endoscopies and multiple imaging modalities. Our patient's history of Crohn's disease likely delayed his diagnosis due to the considerable overlap in symptomatology. Such delays in the detection of obstructive masses can lead to prolonged discomfort and potentially fatal complications for patients.

## Data Availability

Data supporting this research article are available from the corresponding author or first author on reasonable request.
